# Selection and Biosensor Application of Aptamers for Small Molecules

**DOI:** 10.3389/fchem.2016.00025

**Published:** 2016-06-15

**Authors:** Franziska Pfeiffer, Günter Mayer

**Affiliations:** Department of Chemical Biology, Life and Medical Sciences Institute, University of BonnBonn, Germany

**Keywords:** aptamer, small molecule, sensor, aptasensor, oligonucleotide

## Abstract

Small molecules play a major role in the human body and as drugs, toxins, and chemicals. Tools to detect and quantify them are therefore in high demand. This review will give an overview about aptamers interacting with small molecules and their selection. We discuss the current state of the field, including advantages as well as problems associated with their use and possible solutions to tackle these. We then discuss different kinds of small molecule aptamer-based sensors described in literature and their applications, ranging from detecting drinking water contaminations to RNA imaging.

## Introduction

### State of the art

It has been 25 years since the invention of SELEX (systematic evolution of ligands by exponential enrichment) by Tuerk and Gold (1990) and Ellington and Szostak (1990), and likewise for the generation of ribozymes by Joyce (1989). Since then, aptamers have been generated for a multitude of targets such as proteins (Tuerk et al., 1992), small molecules (Jenison et al., 1994), ions (Hofmann et al., 1997), cells (Mayer et al., 2010), and even viruses (London et al., 2015). The year 2004 saw the introduction of the first aptamer-based drug into the pharmaceutical market: Macugen, a highly modified RNA aptamer that binds to the vascular endothelial growth factor (VEGF) and is used to treat age-related macula degeneration (Gragoudas et al., 2004; Ng et al., 2006). Several additional aptamers are in clinical trials, but so far, none has been approved (Sundaram et al., 2013).

Following the discovery of the RNA interference (RNAi) phenomena in 1998 (Fire et al., 1998), synthetic small interfering RNAs (siRNAs) were described as effective tools to induce RNAi in plants by Hamilton and Baulcombe (1999). Soon after, this effect was also achieved in mammalian cells (Elbashir et al., 2001). Although siRNA was discovered almost a decade after the onset of aptamers, they are far more widely accepted as research tools^1^. As both siRNA and aptamers are based on synthetic nucleic acids, one wonders what makes siRNA far more popular.

The most obvious difference lies in the structure. While siRNAs are double-stranded (Elbashir et al., 2001) molecules, whose sequence composition can be designed with regard to the targeted messenger RNA (mRNA), aptamers consist of single-stranded oligonucleotides that have to fold into a specific three-dimensional conformation in order to be able to bind their target molecules (Ellington and Szostak, 1990). This folding depends on temperature and environmental conditions (e.g., buffer, intracellular, extracellular, plasma). It is therefore essential to apply the conditions required for the planned application during the selection process or to reevaluate aptamer binding under the final conditions (Cho et al., 2009; Li N. et al., 2009). Additionally, aptamers have to be identified by an iterative process, for which purified and homogenous targets are required. This is a time consuming and not always successful procedure, limiting their applicability and widespread use to specialized laboratories. RNA aptamers in particular are also prone to nuclease digestion (White et al., 2000). Due to the belief that RNA is structurally more diverse than single-stranded DNA and hence the better choice for aptamer selections (Harada and Frankel, 1995; Lakhin et al., 2013), the majority of aptamers selected for small molecules until 2007 consist of RNA (McKeague and Derosa, 2012). Since then, selections yielding DNA aptamers have been on the rise (Table 1 and McKeague and Derosa, 2012), given their higher stability and lower synthesis cost.

**Table 1:** **Aptamers for small molecule targets described in literature from 2012 to 2015**.

**Target**	**Binding affinity [K_*d*_ (nM)]**	**Chemistry**	**Year**	**References**
Digoxin	8–44	DNA	2012	Kiani et al., 2012
Kanamycin A	3900–24000	DNA	2012	Stoltenburg et al., 2012
Lysergamine	44–499	DNA	2012	Rouah-Martin et al., 2012
Sulfadimethoxine	84–150	DNA	2012	Song et al., 2012b
Trinitrotoluene (TNT)	not reported	DNA	2012	Ho et al., 2012
Abscisic acid	800–1000	DNA	2013	Grozio et al., 2013
Codeine	910	DNA	2013	Huang et al., 2013
N-acetylneuraminic acid	1.4	RNA	2013	Cho et al., 2013
N-glycolylneuraminic acid	6.7	DNA	2013	Gong et al., 2013
N-methyl-mesoporphyrin	1200–43000	DNA	2013	Yang and Bowser, 2013
Okadaic acid	77–983	DNA	2013	Eissa et al., 2013
Saxitoxin	133	DNA	2013, 2015	Handy et al., 2013; Zheng et al., 2015
Streptomycin	199–341	DNA	2013	Zhou et al., 2013
Xanthine	4200–18100	DNA	2013	Bae et al., 2013
Zearalenone	41	DNA	2013	Chen et al., 2013
17ß-Estradiol	900–124000	DNA	2014	Vanschoenbeek et al., 2015
17ß-estradiol	50	DNA	2014	Alsager et al., 2014
Aflatoxin B_1_	96–221	DNA	2014	Malhotra et al., 2014
Aflatoxin B_1_	650	DNA	2014	Zhu et al., 2014
Aflatoxin M_1_	35–1515	DNA	2014	Malhotra et al., 2014
Azobenzene-peptide	not reported	RNA	2014	Hayashi and Nakatani, 2014
Bromacil	9.6	DNA	2014	Williams et al., 2014b
Cortisol	7000–16000	DNA	2014	Martin et al., 2014
Cylindrospermopsin	57–180	DNA	2014	Elshafey et al., 2014
Danofloxacin	3–7.7	2′F-RNA	2014	Han et al., 2014
DFHBI-IT^A^	360	RNA	2014	Filonov et al., 2014
Kanamycin A	2800–163000	DNA	2014	Nikolaus and Strehlitz, 2014
Ketamine	590–660	DNA	2014	Sun M.Q. et al., 2014
Ochratoxin A	110–370	DNA	2014	McKeague et al., 2014
Oxytetracycline	4.9	DNA	2014	Kim et al., 2014
Progesterone	10–133	DNA	2014	Contreras Jiménez et al., 2015
Sphingosine-1-phosphate	4.3	L-RNA	2014	Purschke et al., 2014
T-2 toxin	20.8	DNA	2014	Chen et al., 2014
Thiazole orange	3.2	RNA	2014	Dolgosheina et al., 2014
Trinitrotoluene (TNT)	100	DNA	2014	Priyanka et al., 2014
17ß-estradiol	600	DNA	2015	Akki et al., 2015
17α-ethynylestradiol	500–1000	DNA	2015	Akki et al., 2015
Anatoxin-a	15–81	DNA	2015	Elshafey et al., 2015
Brevetoxin-2	42	DNA	2015	Eissa et al., 2015
DFHBI	not reported^B^	RNA	2015	Zou et al., 2015
Dinitroaniline	100	RNA	2015	Arora et al., 2015
Melamine	510	DNA	2015	Gu et al., 2015
Quinolone	0.1–56.9	DNA	2015	Reinemann et al., 2016

*Given are the K_d_-values reported in the respective reference. For aptamers described between 1990 and 2012, refer to McKeague and Derosa (2012)*.

A *(Z)-4-(3,5-difluoro-4-hydroxybenzylidene)-1,2-dimethyl-1H-imidazol-5(4H)-one*.

B *Selection was performed in bacterial cells to select for high fluorescence, not affinity*.

In addition, siRNA hijacks an endogenous mechanism, which evolved naturally over millions of years, for microRNA maturation and functioning to cause the repression, and degradation of the targeted mRNA (Ozcan et al., 2015). Aptamers, in contrast, do not benefit from such a cell-inherent mechanism and must therefore include all functionalities necessary for their activity within their sequence and folded structure.

Consequently, siRNAs seem to be easier to use than aptamers. However, aptamers offer a much broader range of applications. Furthermore, aptamer binding to e.g., a protein sub-domain leads to a “true” protein inhibition and facilitates functional analysis without gene or mRNA knockdown (as caused by siRNA), which would result in the loss of the entire protein.

Regardless of the difficulties in working with aptamers as elaborated above, aptamers have been hailed as an alternative for antibodies because of their higher stability, lower production cost, and the ease of modification (Ruigrok et al., 2011). There have been numerous reports on their use for cancer cell targeting (Pofahl et al., 2014); as tools for medical therapeutics (Sundaram et al., 2013) or diagnostics (Wan et al., 2010), and biosensors (Potyrailo et al., 1998), which will be the focus of this review.

A search on Pubmed for the terms “aptamer” and “sensor” gives 499 (02.01.2016) hits, proving that a multitude of ideas for the use of aptamers as sensors exist. Many of them are for small molecule targets, which include toxins (McKeague et al., 2014), food and environmental contaminants such as plastic remnants (Lee et al., 2011) and antibiotics (Stoltenburg et al., 2012), explosives (Ehrentreich-Förster et al., 2008), drugs (Ferguson et al., 2013), and protein cofactors (Strack et al., 2014). Nonetheless, to the best of our knowledge, commercially available are only two kits for the quantification of thrombin^2^ and activated protein C (APC)^3^, both of which are protein targets.

Apart from the importance of folding mentioned previously, many aptamers binding to small molecules possess affinities in the micrometer range (McKeague and Derosa, 2012), which means that sensors based on them might not reach the necessary sensitivity. Additionally, information on aptamer structure is often needed for sensor design (Liu et al., 2009), yet remains rare. Out of the >100 aptamers published that bind to small molecules, X-ray- or NMR (nuclear magnetic resonance)-structures are available for only 15 of them (Table 2). In contrast, a high number of riboswitch structures have been solved^4^, demonstrating that the required knowledge is available. It just needs to be implemented for small molecule aptamers and their ligands.

**Table 2:** **NMR and X-ray crystallography structures of aptamers for small molecules that have been deposited into the Protein Data Bank (PDB)**.

**Target**	**PDB-ID**	**Chemistry**	**Technique**	**Year**	**References**
AMP	1AM0	RNA	NMR	1996	Jiang et al., 1996
AMP	1RAW	RNA	NMR	1996	Dieckmann et al., 1996
AMP	1AW4	DNA	NMR	1997	Lin et al., 1997
Argininamide	1DB6	DNA	NMR	2000	Robertson et al., 2000
Arginine	1KOC	RNA	NMR	1996	Yang et al., 1996
Arginine	2ARG	DNA	NMR	1998	Lin et al., 1998
Biotin	1F27	RNA	X-ray	2000	Nix et al., 2000
Citrulline	1KOD	RNA	NMR	1996	Yang et al., 1996
Cyanocobalamin	1DDY	RNA	X-ray	2000	Sussman et al., 2000
Cyanocobalamin	1ET4	RNA	X-ray	2000	Sussman and Wilson, 2000
DFHBI	4Q9Q, 4Q9R, 4KZD, and 4KZE	RNA	X-ray	2014	Huang et al., 2014
DFHBI	4TS2 and 4TS0	RNA	X-ray	2014	Warner et al., 2014
FMN	1FMN	RNA	NMR	1996	Fan et al., 1996
GTP	2AU4	RNA	NMR	2006	Carothers et al., 2006
Malachite Green	1F1T	RNA	X-ray	2000	Baugh et al., 2000
Malachite Green	1Q8N	RNA	NMR	2004	Flinders et al., 2004
Neomycin B	1NEM	RNA	NMR	1999	Jiang et al., 1999
Streptomycin	1NTB and 1NTA	RNA	X-ray	2003	Tereshko et al., 2003
Tetracycline	3EGZ	RNA	X-ray	2008	Xiao et al., 2008
Theophylline	1EHT	RNA	NMR	1997	Zimmermann et al., 1997
Theophylline	1O15	RNA	NMR	2003	Clore and Kuszewski, 2003
Tobramycin	1TOB	RNA	NMR	1997	Jiang et al., 1997
Tobramycin	2TOB	RNA	NMR	1998	Jiang and Patel, 1998

*AMP, adenosine monophosphate*.

Finally, sensors that work in proof-of-concept studies, mainly performed in academia, do not necessarily match the conditions, the statistical relevance, the compatibility with routine equipment found in e.g., clinical diagnostic departments, and the scale required for an industrial and/or clinical application. Thus, time-consuming optimizations need to be done prior to a possible market introduction.

### Where we want to be and what we need to get there

Instead of vastly being an academic point of interest, we would like aptamers to reach their potential as real alternative affinity tools—because we firmly believe that this is achievable. This does not only mean that they should be more widely used as research tools in academia. It includes increased acceptance and commercialization by the industry. Table 3 gives an overview of companies already involved in aptamer commercialization. Furthermore, in the last couple of years, antibodies as the most widely used affinity tool have been questioned due to problems with variability between different batches and cross reactivity (Baker, 2015). Maybe it is the right time for aptamers to finally enter the stage.

**Table 3:** **Companies involved in aptamer commercialization; offering aptamers, their generation, and/or application**.

**Company**	**Location**	**Website**	**Founded**
Alpha Diagnostic International	United States	http://www.4adi.com	1993
AMBiotech	United States	http://am-biotech.com	2009
AptaBiosciences	Singapore	http://www.aptabiosciences.com	2013
Apta Biotherapeutics	South Korea	http://www.aptabio.com	2013
Aptagen	United States	http://www.aptagen.com	2004
Aptahem	Sweden	http://aptahem.com	2014
Aptamer Group^A^	United Kingdom	http://www.aptamergroup.co.uk	2008
Aptamatrix	United States	http://www.aptamatrix.com	2003
Apterna	United Kingdom	http://apterna.com	2011
Aptitude Medical	United States	http://www.aptitudemedical.com	2011
Aptus Biotech^B^	Spain	http://www.aptusbiotech.com	2010
Aptamer Sciences	South Korea	http://www.aptsci.com	2011
Basepair Technologies	United States	http://www.basepairbio.com	2011
Berlin Cures GmbH	Germany	http://berlincures.de	2014
CD Genomics	United States	http://www.cd-genomics.com	2004
Ice9Biotechnologies	United States	http://www.iceninebio.com	2009
Neoventures Biotechnology	Canada	http://www.neoventures.ca	2002
Novaptech	France	http://www.novaptech.com	2008
NOXXON Pharma Ag	Germany	http://www.noxxon.com	1998
Ribomic	Japan	http://www.ribomic.com	2003
SomaLogic	United States	http://www.somalogic.com	2000

A *also Aptasol, AptaDx, AptaRx, and Aptasort*.

B *also Aptatargets—http://www.aptatargets.com*.

In order to convince both academia and industry that aptamers are trustworthy affinity tools, they need to demonstrate trustworthiness. That is, they should function as described in the publications. Publications have to include exact (and understandable as well as easily accessible) information on the identified sequence, folding conditions, buffer requirements, target handling protocols, and temperature applied. If at all possible, a different technique than the one used for the selection should be used to determine the aptamer-target dissociation constant and its specificity. This is especially important for aptamers binding to small molecules, which are almost always selected to bind to the immobilized compound during the selection process. Thus, binding in solution has to be proven after enrichment. Using two different methods for the affinity measurements further means that the range of obtained *K*_d_-values will be more trustworthy as McKeague et al. recently showed that the affinity can differ vastly depending on the technique used (McKeague et al., 2015). The best way to proof binding of new aptamers would obviously be to have them verified in a reference laboratory, an idea that has previously been discussed by Famulok and Mayer (2014). For example, a recently published aptamer for a protein target went into the right direction by portraying affinity measurements performed with three different techniques in three independent laboratories (Jauset Rubio et al., 2016). In addition to these issues concerning affinity determination, time and effort should be taken to elucidate aptamer structure and the binding region—apart from simplifying sensor design (Liu et al., 2009), shortening the aptamer to the motif necessary for binding not only decreases its production cost, but can also improve its affinity (Sassanfar and Szostak, 1993; Kwon et al., 2014).

## Aptamers binding to small molecules

### Problems associated with the selection of aptamers binding to small molecules

Selecting aptamers interacting with small molecules is accompanied by some very specific problems that do not occur during selections using proteins and other target molecules. First of all, due to their small size, the functional groups and therefore the possible interactions between the ligand and the aptamer are much more limited. If, as in most cases, the selection is not performed with the target in solution, one of its functional groups will have to be used for the immobilization, thereby further decreasing the amount of possible interactions with a future aptamer. Moreover, the immobilization may generate a novel epitope that is required for aptamer interaction. As aptamers rely on electrostatic, H-bonds, hydrophilic or π-π-stacking interactions for binding, a limited amount of functional groups does not only influence the probability of being able to select an aptamer at all, but might also mean that the selected aptamer binds worse to the target in solution (Wilson and Szostak, 1998), if at all (if the now free functional group disturbs aptamer binding). The aptamer's ability to distinguish between closely related molecules that only differ at the immobilization site might also be negatively affected (Mannironi et al., 1997).

For protein targets, the selection with nucleobase-modified nucleic acid libraries has recently been shown to increase the success rate of selections immensely, most likely because of the additional interaction possibilities introduced with the modification (Gold et al., 2010). A similar, but modular approach where the modification could be freely chosen was also published recently (Tolle et al., 2015). As stated above, the limited number of interaction possibilities is often a problem in regard to using small molecule targets. Transferring these techniques to the selection of aptamers binding to small molecules might enhance the chances for successful selections even for difficult targets.

Affinity elution with a high target concentration is usually used during the selection process to set the stage for aptamers that also recognize the target in solution (Sassanfar and Szostak, 1993). Depending on the solubility of the target molecule, these high concentrations might not be achievable, leaving binding to the non-immobilized target up to chance. In addition, detecting binding in solution at all might be a problem if the target's solubility in the mostly aqueous buffers used for the selection and binding assays is too low compared to the aptamer's affinity.

### Selection and immobilization strategies

Even though a number of different selection techniques have been established in the last years (McKeague and Derosa, 2012), most of them are not adaptable for small molecules due to their small size. Some selection techniques that evade the need for target immobilization have been tested with small molecules and will be explained here, but in most cases, the target is still required to be immobilized.

Capillary electrophoresis-SELEX (CE-SELEX) in particular has been shown to lead to a much stronger separation between bound and non-bound sequences, the decisive step of every aptamer selection process. It thereby decreases the amount of selection cycles needed for successful enrichment of binding species (1–4 instead of 8–12 with conventional partitioning techniques). CE-SELEX uses the different migration of free nucleic acids in the capillary in contrast to those bound to the protein target. Obviously, the small size of small molecules does not lead to a large change in migration. Hitherto, Yang et al. were the only group to successfully perform a CE-SELEX with a small molecule target, porphyrin, which has a molecular weight of 580 Da. However, they selected blindly, choosing the fractions just before the normal nucleic acid peak without the aid of having a visible peak to collect as is normally the case during protein CE-selections (Yang and Bowser, 2013). Whether this approach is applicable to many small molecules remains to be determined in future experiments.

A different technique to select aptamers without the need for target immobilization is the capture-SELEX approach. Here, beads are modified with a capture oligodeoxynucleotide complementary to a short docking sequence embedded in the random region of the nucleic acid library. After annealing of the entire DNA or RNA library to the complementary sequence on the beads, the beads are incubated with the target of interest. Only sequences binding to the target and thereby undergoing conformational changes that lead to detachment from the bead will be selected. These sequences can be amplified and used for the subsequent selection cycles (Nutiu and Li, 2005; Stoltenburg et al., 2012). While the selection was performed with kanamycin A in solution, this technique needed neither less selection cycles (13 were performed) nor did it result in aptamers with higher affinities than conventional selection approaches using the same target (Song et al., 2011). This means the capture-SELEX approach is probably more useful for selections where immobilization of the target is not easily feasible.

When it comes to immobilizing the target, the choice is mainly between two different matrixes: magnetic beads or agarose. Both allow a variety of chemistries for the coupling reaction (Table 4).

**Table 4:** **Commercially available, chemically modified matrices for target immobilization for small molecule aptamer selections**.

**Chemical moiety of ligand**	**Matrix chemistry**	**Magnetic bead^A^^,^^E^**	**Agarose**
COOH CHO	hydrazide		Adipic acid dihydrazide Agarose^C^
COOH CHO (EAH Sepharose only)	amine	M-270 Amine	EAH Sepharose 4B^B^ Affi-Gel 102 Gel^D^ CH sepharose 4B^C^ CarboxyLink Coupling Resin^E^
NH_2_	aldehyde		AminoLink Plus Coupling Resin^E^ AminoLink Coupling Resin^E^
NH_2_	cyanogen bromide		CNBr-Activated Sepharose 4B^B^ CNBr-Activated Sepharose 6 MB^B^
NH_2_	N-hydroxy succinimide (NHS)		NHS-Activated Sepharose E Fast Flow^B^ Affi-Gel 10^D^ Affi-Gel 15^D^ Pierce NHS-Activated Agarose^E^
NH_2_ N-nucleophiles	carbonyl diimidazole		Pierce CDI-activated Agarose Resin^E^
NH_2_ SH	tosylactivated	M-280 Tosylactivated MyOne Tosylactivated	
NH_2_ SH (Dynabeads only)	carboxylic acid	M-270 Carboxylic Acid MyOne Carboxylic Acid	ECH Sepharose 4B^B^
NH_2_ SH OH (Sepharose only)	epoxy	M-270 Epoxy	Epoxy-Activated Sepharose 6B^B^
SH	iodacetyl		SulfoLink Coupling Resin^E^
SH heavy metal ions alkyl and aryl halides addition to C=O C=C N=N	thiol		Activated Thiol Sepharose 4B^B^ Thiopropyl Sepharose 6B^B^

*Manufacturers are indicated by superscripted capitals*.

A *brand name Dynabeads*.

B *GE Healthcare*.

C *Sigma*.

D *Bio-Rad*.

E *ThermoFisher Scientific*.

Magnetic beads simplify the selection process as separation between bound and non-bound sequences can be facilitated using a magnet. In addition, they can be analyzed using flow cytometry, which also represents one possibility for binding analysis (i.e., using fluorescently-labeled DNA or RNA; Tolle et al., 2015) and to determine coupling of the target to the beads (e.g., using fluorescently-labeled target-binding antibodies).

Agarose is still the most widely used matrix for small molecule aptamer selections. It requires a column with a membrane to separate bound and non-bound sequences. Agarose can be used for techniques such as isocratic affinity elution (Huizenga and Szostak, 1995) and can immobilize the target to a much higher density than magnetic beads^5^. This enables the selection of aptamers with lower affinities if none with higher affinity are available. Seeing the interest in photo-regulation of aptamer activity (Young and Deiters, 2008), it might also be worth pointing out that agarose is a clear medium that can be irradiated with a wavelength of interest.

## Small molecule aptasensors

The gold standard for the detection of small molecules is mass spectrometry (Sudsakorn et al., 2011). However, it requires expensive and large instrumentation as well as a high degree of training to be used correctly. Alternative analysis tools such as antibody- and aptamer-based sensors, albeit lacking the sensitivity and selectivity of mass spectrometry, have become popular as they can be used with little training (instruction leaflets are normally sufficient) and at the site of sampling. This, in addition to the fact that the sensors themselves are cheaper, means that altogether, the sample analysis is far less expensive.

### Sensor types

**Figure 1:**
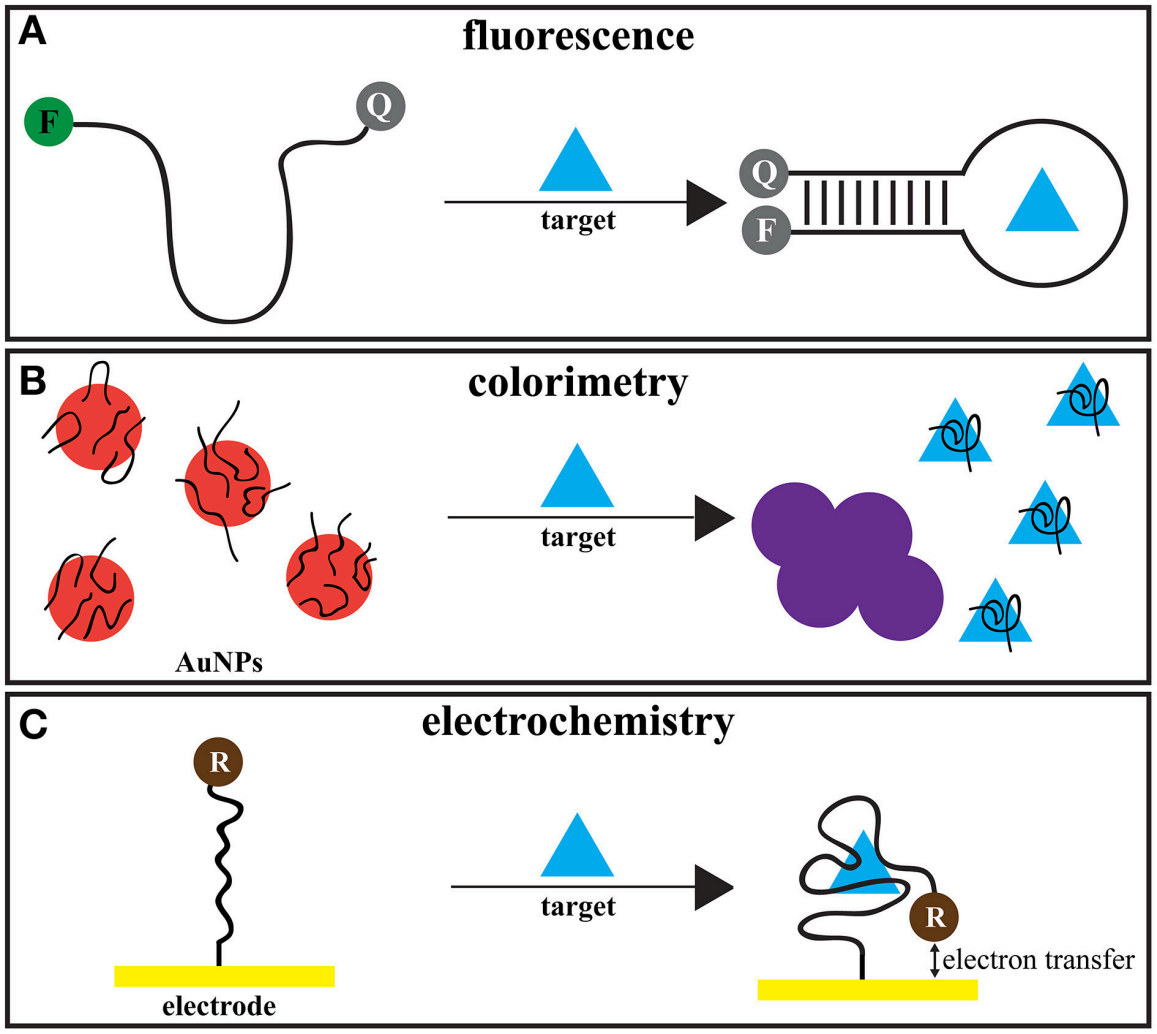
**Small molecule aptasensor types**. Depicted is an example for a sensor for the three most widely used types for small molecule targets. F, fluorophore. Q, quencher. AuNPs, gold nanoparticles. R, redox probe. **(A)** The aptamer is labeled with a fluorophore and an appropriate quencher. Upon binding to the target, the conformational change of the aptamer brings fluorophore and quencher into close contact, thereby quenching the fluorescence. **(B)** The aptamer is unspecifically absorbed onto the surface of AuNPs and thus prevents their aggregation. Upon binding to the target, the AuNPs aggregate. This leads to a visible color change from red to blue. **(C)** The aptamer is immobilized onto an electrode and labeled with a redox probe. The conformational change upon target binding brings the probe close enough to the electrode to allow electron transfer and thus, an electrochemical readout.

Sensors based on aptamers, also called aptasensors, come with a variety of readout mechanisms. Since the publication of the enzyme-linked oligonucleotide assay in 1996 by Drolet et al. (1996) and the first aptamer-based sensor in 1998 (Potyrailo et al., 1998), fluorescence (Stojanovic et al., 2001), colorimetry (Song et al., 2011), electrochemistry (Ferapontova et al., 2008), luminescence (Leung et al., 2013), mass sensitivity (Cappi et al., 2015), and amplification of nucleic acids (Cho et al., 2005) have been used as readout formats. An overview of the three most widely used types of small molecule aptasensors is depicted in Figure 1.

Han et al. reviewed strategies for the design of aptamer-based biosensors, dividing them into target-induced structure switching, target-induced dissociation/displacement, sandwich or sandwich-like and competitive replacement based (Han et al., 2010). Following this categorization, we will elucidate what kind of prior knowledge about and properties of the aptamer are needed for the different sensor design strategies.

Binding of small molecule aptamers to their ligand is frequently accompanied by a conformational change within the aptamer's structure (McKeague and Derosa, 2012). Therefore, structure-switching sensors are quite common, but need prior knowledge about the aptamer structure for a rational design-based approach (Osypova et al., 2015). To circumvent laborious structure analysis, a technique for the *de novo* selection of structure-switching aptamers has also been published (Nutiu and Li, 2005). In contrast, for most dissociation and displacement sensors, sequence information is sufficient (Yoshizumi et al., 2008). Some more elaborate approaches also consider structural information on e.g., stem formation to fine-tune the target-induced displacement strategy (Liu et al., 2006).

Due to the size of small molecules, traditional sandwich assays that require binding of multiple recognition elements to the target (Fang et al., 2008) are not possible (Han et al., 2010). A way to circumvent this would be the selection of an aptamer that does not recognize the target or another aptamer alone, but only the aptamer-target-complex. Sandwich assays based on structure switching and dissociation/displacement have been implemented (Zhang et al., 2008).

Competitive replacement assays normally rely on binding of the aptamer to the immobilized target and, upon addition of the sample, binding of the aptamer to the target in solution, thereby being displaced from the immobilized one (de-los-Santos-Alvarez et al., 2007). This requires target immobilization and, most importantly, an affinity for the target in solution that is at least as high as the one for the immobilized target, as the latter is present at a high local concentration on the solid phase.

### Sensor applications

**Figure 2:**
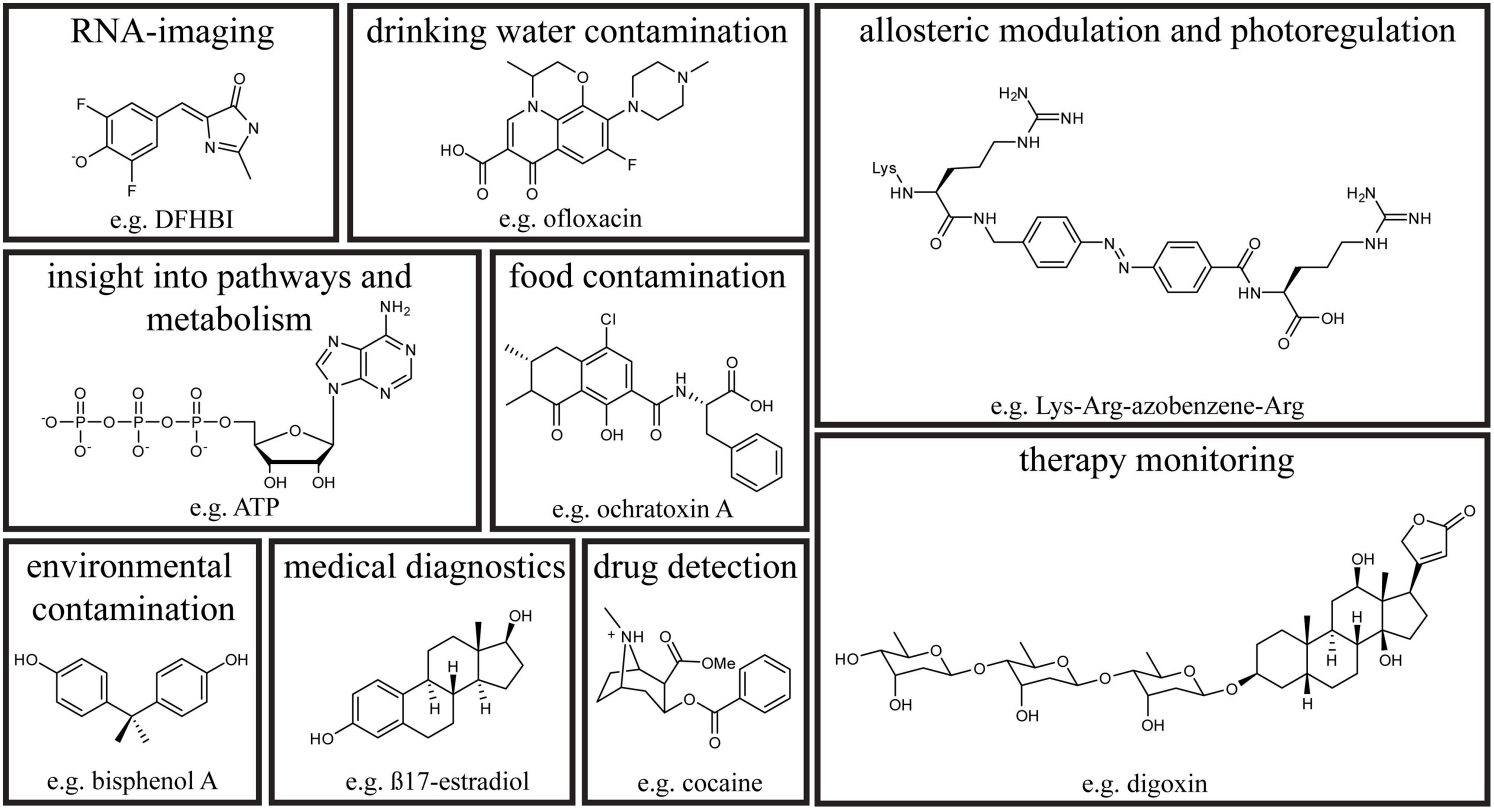
**Small molecule aptasensor application fields**. Depicted is an example for a small molecule target of the respective field including its chemical structure.

An overview of aptasensor application fields and the structure of a target for each field is given in Figure 2.

#### Food and drinking water contaminants and environmental pollutants

Food and drinking water are tightly controlled to prevent the contamination with harmful substances or bacteria. In order to enforce this control, the detection of contaminants and, if possible, their removal is of utmost importance (García-Cañas et al., 2012). Although obviously part of the problem, pathogens, allergens and large protein toxins such as ricin are not small molecules and will therefore not be discussed here. They are included in a review about aptamer-based sensors for food control from 2013 by Amaya-González et al. (2013).

##### Small molecule toxins

Small molecule toxins that aptamers have been selected for can be divided into three classes: mycotoxins and cyanotoxins, produced by fungi and water inhabiting cyanobacteria, respectively, and toxins from dinoflagellates, which upon ingestion lead to shellfish poisoning.

Mycotoxins are fungal toxins that contaminate grain and grain-derived products such as beer and milk, but can also be found e.g., in coffee. They can cause a variety of diseases and some are known to be highly carcinogenic (Richard, 2007; Li P. et al., 2009). Due to this, the Food and Agricultural Organization of the United Nations classifies them as a risk for human and animal health^6^.

The most frequently used aptamer recognizing mycotoxins is the one for ochratoxin A. As a matter of fact, so many sensors were based on the aptamer that they were reviewed on their own by Rhouati et al. (2013). Further reviews also deal with ochratoxin A aptasensors amongst others (Amaya-González et al., 2013; Vidal et al., 2013; Ha, 2015), which is why we will be focusing on aptasensors available for the other mycotoxins.

Aptasensors for the detection of aflatoxin B_1_ and M_1_ exist in combination with a plethora of techniques: qPCR (Guo et al., 2014), split aptazymes (Seok et al., 2015), and dipstick assays (a more detailed description about the workflow for these easy to use sensors can be found in Section Drugs of Abuse Detection; Shim et al., 2014) as well as the more widely spread electrochemical (Nguyen et al., 2013; Istamboulié et al., 2016) and colorimetric (Malhotra et al., 2014), fluorescent (Wang B. et al., 2015) and chemiluminescent (Hosseini et al., 2015) gold nanoparticle based sensors. Some sensors were shown to work in spiked or real samples (Guo et al., 2014; Seok et al., 2015; Istamboulié et al., 2016) and with results similar to those obtained with the normally used immunoassays (Shim et al., 2014).

Other mycotoxins, for which aptamer-based sensors have been published, are fumonisin, lysergamine, and T-2 toxin. They managed to detect T-2 toxin in beer (Chen et al., 2014) and small ergot alkaloids, which were structurally similar to lysergamine, in flour (Rouah-Martin et al., 2012). An aptamer for zearalenone was selected, but has not been implemented in a sensor yet (Chen et al., 2013).

As one fungus can produce several mycotoxins and the same mycotoxin can be produced by several fungi species, being able to detect if a sample is contaminated and with which toxins is of high importance—and the simultaneous detection of several toxins would help to achieve this goal (Turner et al., 2015). Toward this objective, two fluorescence-based aptasensors capable of simultaneously detecting fumonisin B_1_ and ochratoxin A have been generated, with detection limits reaching 0.16 and 0.25 pg/ml, respectively. Both were used for the detection of the mycotoxins in real samples and worked as well as the usually used ELISA (enzyme-linked immunosorbent assay) (Wu S. et al., 2012; Yue et al., 2014).

Cyanotoxins are hepatotoxic and can result in sickness or even death of the exposed person or animal (Azevedo et al., 2002). One of them is microcystin-LR, a cyclic heptapeptide with a molecular mass of nearly 1000 Da. A sandwich assay combining an HRP (horse radish peroxidase)-labeled antibody and an aptamer (Xiang et al., 2014) as well as two aptamer-based electrochemical sensors managed to detect microcystin-LR in spiked water (Lin et al., 2013) or even fish samples (Eissa et al., 2014). A different, colorimetric sensor needed only 5 min analysis time (Wang F. et al., 2015), qualifying it for on-site usage. Efforts have also been undertaken to remove microcystin-LR from drinking water using the aptamer immobilized on graphene oxide nanosheets (Hu et al., 2012). Remarkably, all sensors described here reach the 1 μg/L limit set by the World Health Organization.

Aptamers for two additional, much smaller cyanotoxins, anatoxin-a (a neurotoxin also called “very fast death factor”) and cylindrospermopsin (an alkaloid) have been selected recently. Moreover, both have been implemented in structure-switching electrochemical biosensors (Elshafey et al., 2014, 2015; Zhao Z. et al., 2015).

Toxins invoking shellfish poisoning have also been addressed by SELEX recently: Aptamers for the neurotoxin saxitoxin have been selected (Handy et al., 2013) and improved (Zheng et al., 2015), but have not been used as sensing elements yet. Brevetoxin-2 (a neurotoxin) and okadaic acid (causing diarrhea) could both be detected in spiked shellfish using electrochemical biosensors developed by Eissa et al. (2013, 2015).

##### Hormones

High doses of the female sexual hormones progesterone and 17ß-estradiol disrupt the endocrine system in both men and women. As only limited amounts are actually absorbed by the human body, the rest ends up in the water and thereby in food and environment. An electrochemical sensor for the detection of such progesterone contamination of drinking water was published by Contreras Jiménez et al. (2015).

Several different aptamers recognizing 17ß-estradiol are known (Kim et al., 2007; Alsager et al., 2014; Akki et al., 2015) and have been used in sensors to detect estradiol in urine (Lin et al., 2012; Huang et al., 2015; Zhu B. et al., 2015), water (Yildirim et al., 2012; Fan et al., 2014, 2015; Akki et al., 2015), and both matrixes (Alsager et al., 2015). A colorimetric sensor based on gold nanoparticles was able to visualize physiological concentrations of estradiol in urine—without the need for expensive readout technologies (Soh et al., 2015). Huang et al. published an overview of estradiol sensors based on both antibodies and aptamers. At least for those listed, aptamer-based sensors show a remarkably low limit of detection (Huang et al., 2015).

##### Pollutants of industrial origin

Natural toxins and hormones are not the only problem when it comes to food, water, and environmental pollution. Contamination with toxic molecules of industrial origin is just as common and ever increasing due to the rising production demands of industrial goods.

One of these pollutants are polychlorinated biphenyls (PCBs), which were used as lubricants and coolants in industry and have, due to their high stability and lipophilic properties, accumulated in nature (Mehta et al., 2012). As they are toxic and carcinogenic, their production was banned more than 10 years ago (Porta and Zumeta, 2002), but they are still present in the environment and, therefore, specific detection devices are needed. Due to their chemical heterogeneity (209 different PCBs are known) (Mehta et al., 2012), a perfect sensing device would recognize all of them, which would require reduced aptamer specificity, contrary to usual design. Mehta et al. selected an aptamer that showed binding to six different PCBs, even though the interaction was weaker with those that were not used as targets during the selection process (Mehta et al., 2012). The aptamer was used for the detection of hydroxylated PCBs spiked into blood serum (Pilehvar et al., 2014). It was also implemented in a sensor for the simultaneous detection of chloramphenicol and PCBs in spiked fish samples. Due to the fact that metal ions were used as tracers to determine which of the two targets was being detected, the number of targets that can be analyzed in parallel is limited by the number of applicable ions (Yan et al., 2015).

Xu et al. chose PCB77, one of the most toxic PCBs, as target for their selection and for the design of a gold nanoparticle based sensor. Their aptamer recognized some additional PCBs, but also to a lesser extent than PCB77 (Xu et al., 2012). Two other sensors were build using this aptamer (Lu et al., 2014), one of which managed to achieve a detection limit of 10 nM (Fu et al., 2015), even though the aptamer's affinity was in the micromolar range (Xu et al., 2012), by using surface-enhanced Raman scattering with build-in Ag-nanocrown arrays for signal enhancement (Fu et al., 2015).

Bisphenol A is used in plastic production processes, but has spread from plastic packaging and bottles into food and drinks and is now regarded as an environmental contaminant (World Health Organization and Food and Agriculture Organization of the United Nations, 2011). If ingested, it can bind the estrogen receptor and thereby disrupt the endocrine hormonal pathway. A multitude of aptasensors detecting bisphenol A were successfully used in water (Jo et al., 2011; Ragavan et al., 2013; Kuang et al., 2014; Yildirim et al., 2014; Chung et al., 2015; Wang D. et al., 2015; Zhu et al., 2015a; Chen and Zhou, 2016; Yu et al., 2016), milk (Zhou et al., 2014), and serum samples (Zhu et al., 2015b) and one could be interpreted with the naked eye without the need for costly and heavy instrumentation (Mei et al., 2013). Several publications give overviews about detection limits and ranges for different bisphenol A sensors (Kuang et al., 2014; Zhu et al., 2015b; Yu et al., 2016).

Melamine is usually used in industrial production processes, but its high nitrogen content has led to its use as an adulterant in food products, where it seemingly increases the protein content. As it has a high renal toxicity, its detection as food contamination is strongly recommended (Mauer et al., 2009). An aptasensor using a personal glucose meter for readout was developed: Magnetic beads and invertase were both conjugated to DNA strands, which are complementary to the aptamer sequence. Upon addition of melamine, the aptamer changes its structure and dissociates from the complementary strands, enabling magnetic separation of the beads and invertase. The enzyme then converts sucrose into glucose, which can be quantified with a personal glucose meter. The sensor worked in milk with detection limits lower than required by the US Food and Drug Administration (FDA) (Gu et al., 2015).

Methylenedinitroaniline (MDA) is a widely used industrial chemical for e.g., the production of plastics and a thermal degradation product of polyurethanes. It is known to damage DNA and suspected to be carcinogenic. Apart from working or living in the vicinity of a factory, MDA set free from polyurethane in dialysis machines after sterilization has also led to exposure. An aptamer has been selected against MDA, but no sensors based on it have been published yet (Brockstedt et al., 2004).

##### Drugs

Antibiotics are used in both human and veterinary medicine, but also in high amounts in industrial livestock farming and agriculture. Due to the large amounts used, they are a problem for environmental, water, and food cleanliness. Aptamers against tetracycline (Zhang et al., 2010; Luo et al., 2014a; Wang S. et al., 2014, 2015; Ramezani et al., 2015), oxytetracycline (Kim et al., 2014), kanamycin A (Sun X. et al., 2014; Xing et al., 2015), chloramphenicol (Pilehvar et al., 2012; Alibolandi et al., 2015), ampicillin (Song et al., 2012a), sulfadimethoxine (Song et al., 2012b), and streptomycin (Zhou et al., 2013; Danesh et al., 2016; Emrani et al., 2016) have been used in sensors and could detect their target antibiotic in milk or honey. For two quinolone antibiotics, fluoroquinolone (Reinemann et al., 2016), and danofloxacin (Han et al., 2014), aptamers have been selected, but no sensors have been published yet.

Although originally a dye and used as such in biological applications (Kolpashchikov, 2005), malachite green is used in aquaculture because of its antifungal and -parasitic properties. Due to its suspected toxicity, an upper limit for its concentration in fish has been set. An aptasensor reaching this limit of 2 μg/kg in fish samples was published (Stead et al., 2010). In addition, a sensor combining the detection of malachite green and chloramphenicol, which is also often used in aquaculture, was applied for the simultaneous quantification of both contaminants in fish samples (Feng et al., 2015).

Apart from the above mentioned antibiotics, the commonly used over-the-counter pain medication diclofenac is also frequently found in the water-cycle and was shown to be harmful to both fish and birds (Joeng et al., 2009). An aptamer recognizing the drug was used for the cleanup of drinking water (Hu et al., 2011), but has not been implemented in a sensor.

##### Pesticides

Due to their large scale application in agriculture, pesticides and herbicides pose a problem when it comes to water and environmental contamination. Due to the large amount of different compounds in use, testing for single contaminants is impractical (Amaya-González et al., 2013). Nevertheless, aptasensors for a number of organophosphorus pesticides were identified (Pang et al., 2014; Bai et al., 2015), two of which worked in spiked food samples (Tang et al., 2016), but one only after an elaborate extraction protocol (Zhang C. et al., 2014). This fact again emphasizes that effective utilization of aptamers for diagnostic purposes may require an optimization of pre-analytical procedures. Additionally, aptamers for the pesticide malathion and herbicide bromacil have been published (Williams et al., 2014a,b). An array of sensors has been implemented for the detection of the insecticide acetamiprid in soil (Shi H. et al., 2013), water (Shi H. et al., 2013; Fei et al., 2015) and food samples (Fan et al., 2013; Guo et al., 2016; Li et al., 2016). A publication by Fei et al. gives an overview of the detection limits and ranges of published sensors for acetamiprid (Fei et al., 2015).

##### Explosives

2,4,6-Trinitrotoluene (TNT) is a commonly used explosive material whose detection is necessary for public safety as well as environmental and water decontamination. Different aptamers against it were selected with biosensing applications in mind: Ehrentreich-Förster et al. were the only ones up to now to use their sensor for the detection of TNT in soil, solvent and water samples (Ehrentreich-Förster et al., 2008); others developed a sandwich assay (Ho et al., 2012), an electrochemical (Priyanka et al., 2014), and a Förster resonance energy transfer (FRET)-based aptasensor, the latter of which combined the use of a TNT-aptamer and an antibody (Sabherwal et al., 2014), but none was tested in real samples.

#### Drugs of abuse detection

Apart from their impact on driving safety and the resulting necessity to test drivers on the roadside as well as after accidents (Horst et al., 2012), workplace drug testing is nowadays widely spread to either deter employees from using drugs or due to safety concerns (Pidd and Roche, 2014). As drug of abuse detection can be performed using several different matrixes (blood or serum, urine, hair, sweat, and oral fluid), sensor design should consider both the way the drug is present in the specific matrix (active drug/metabolite?) and its concentration range—also over time. Care should also be taken regarding potential masking or interfering agents, which could purposefully have been taken by the test person or be normal matrix components. Up to now, cocaine and codeine are the only illicit drugs for which aptamers have been published (Stojanovic et al., 2001; Win et al., 2006; Huang et al., 2013).

Due to its conformational change upon target binding, the cocaine-binding aptamer is a widely used model for sensor development that relies on structure switching (Stojanovic et al., 2001; Chen et al., 2008). Therefore, a variety of sensors with electrochemical (Baker et al., 2006; Du et al., 2010; Wen et al., 2011; Zhao T. et al., 2015), fluorescent (He et al., 2010; Shi Y. et al., 2013) and colorimetric (Du et al., 2011a) readout, which have sensitivities aligning with the concentrations found in real biological samples, has been developed. Some of the sensors were shown to be insusceptible to agents used to mask and cut cocaine (Baker et al., 2006; Wen et al., 2011). A sensor with a pM detection limit was even employed to detect traces of cocaine on banknotes (Cai et al., 2011). A fluorescent sensor enabled readout with the naked eye for concentrations above 8 μM (Wu et al., 2010). He et al. published an overview of the necessary detection times and limits of the different sensing methods (He et al., 2010).

A solution for the problem that most sensors require expensive instrumentation and the execution of a protocol too difficult for non-trained personnel such as police officers testing a driver for drug use was proposed by Liu et al.: They developed a lateral flow test based on gold nanoparticles. After application of the undiluted blood serum, the wicking pad was dipped into buffer to start the migration of the sample and 5 min later, a visible red line proved the presence of cocaine in the sample (Liu et al., 2006).

The parallel detection of multiple drugs of abuse is necessary for both the traffic and workplace drug testing mentioned above. Such multianalyte quantification was possible in real samples (plasma and/or serum) with microfluidic sensors that detected adenosine/ATP and cocaine (Du et al., 2011b; Zhang H. et al., 2014). A paper-based analytic device enabled parallel detection of cocaine, Pb^2+^ and adenosine with the naked eye in urine in only 6 min. Like the lateral flow test described above, a single loading step was sufficient for the analysis, but the sensor did not allow quantification (Wei et al., 2015).

Even though codeine is far less widely used as an illicit drug, aptamer-based sensors have been published, but their effectiveness in biological samples (e.g., urine or blood) has not been shown (Saberian et al., 2011; Huang et al., 2013). A method to extract codeine from urine based on an aptamer has been developed, but it relies on electrospray ionization ion mobility spectrometry for quantification of the extracted codeine (Hashemian et al., 2015).

#### Insights into pathways and metabolism

Many small molecule aptamer targets are important players in cellular and extracellular pathways as well as metabolism. Therefore, sensors able to detect and quantify these molecules are potential research tools that can give insights into a variety of processes and mechanisms in the human body. For many of these targets, intracellular quantification or detection would be most beneficial, but so far this has only been realized for ATP and GTP.

Nucleotides are the building blocks of RNA and DNA and thus also of aptamers. Adenosine and guanosine triphosphate (ATP, GTP), however, also fulfill other important roles in cellular metabolism as cofactors for protein (de-)activation, e.g., by phosphorylation, whereas ATP is also the most important cellular energy source. Simultaneous detection of GTP and ATP was realized by Wang et al. in living cells and in real time using a fluorescent readout format. Their sensor enabled localization of both nucleotides, but not quantification (Wang Y. et al., 2013). Along the same lines, a multitude of sensing techniques was developed for the fluorescent detection of ATP in living cells in general (Tan et al., 2012; Wu C. et al., 2013; Liu et al., 2014; Jia et al., 2015; Qiang et al., 2015; Wang W. et al., 2015) and in lysosomes specifically (Jin et al., 2014). Yi et al. went a step further and used the deeper penetration depth of two-photon microscopy to visualize ATP in zebrafish (Yi et al., 2014). A different approach quantitatively determined ATP in yeast cells. The method was time-resolved and was used to monitor ATP dynamics under different conditions (Özalp et al., 2010). Sensors to determine extracellular ATP have also been published: Cerebral ATP was quantified in rat brain microdialysates (Yu et al., 2015) and a sensor was able to measure ATP secreted from a single taste receptor cell in response to a taste stimulus (Wu C. et al., 2012).

Apart from ATP and GTP, a variety of different molecules could be detected extracellularly using aptasensors: the second messenger cyclic adenosine monophosphate (cAMP; Zhao F. et al., 2015), the cofactor flavin mononucleotide (FMN; Stojanovic and Kolpashchikov, 2004), the neurotransmitter dopamine (Li et al., 2013), the hormone cortisol (Sanghavi et al., 2016), and the lipid sphingosylphosphorylcholine (Horii et al., 2010). All of these sensors could potentially be used to help elucidate the biological functions and processes that these molecules play a role in.

#### Medical diagnostics and therapy monitoring

As mentioned in the section above, small molecules play important roles in the human body as vitamins, hormones, intracellular messengers, metabolites, and cofactors for proteins. As they change upon disease onset and/or progression, the concentration of the respective small molecule can be used as a diagnostic tool.

Vitamin B_12_ (cyanocobalamin) as well as vitamin B_2_ (riboflavin) are essential nutrients that have to be taken up with food. As deficiencies can lead to anemia (Shi Z. et al., 2014) and, in the case of vitamin B_12_, neurological disorders (Selvakumar and Thakur, 2012), quantification is necessary to detect a deficit early on. A colorimetric aptasensor was used to quantify cyanocobalamin in pharmaceutical preparations (Selvakumar and Thakur, 2012) as well as in *Chlorella vulgaris*, a green algae (Kumudha et al., 2015), but not in real samples like urine or blood. In contrast, one of the two aptasensors published for the detection of riboflavin was successfully tested in both urine and food samples (Xu et al., 2013). An aptamer for vitamin B_7_ (biotin) is also available, but has not yet been implemented in a sensor (Wilson et al., 1998).

Dysregulation of hormones can also lead to a multitude of diseases. Aptasensors developed for hormones that might be used for medical diagnostics and therapy monitoring—most predominantly 17ß-estradiol—are described under the section discussing sensors for food and drinking water contaminants and environmental pollutants (Hormones).

Neurotransmitters are fundamental for the regulation of the nervous system. Aptamers for two different neurotransmitters have been selected: acetylcholine (Bruno et al., 2008) and dopamine. While the aptamer for the former has not been used in sensors, abnormalities in dopamine concentrations are indicative for e.g., schizophrenia, Parkinson's and Alzheimer's disease—and detectable in human serum with aptasensors (Park and Paeng, 2011; Liu et al., 2012).

The concentration of free amino acids gives information on the peptide and protein metabolism of a cell and can be changed significantly upon disease. For example, serum tryptophan levels are decreased in patients suffering from depression. Two different aptasensors for tryptophan were developed, one of which could be used in diluted fetal bovine serum (Yang et al., 2015). Histidine deficiency can lead to Parkinson's disease or epilepsy. It has been quantified using aptamers in electrochemical (Liang et al., 2011) and gold nanocrystal assays (Zhengbo et al., 2013), but not in biological samples. The amino acid derivatives tyrosinamide and argininamide, in contrast, could be measured in urine, serum or fetal bovine serum with different aptasensors (Ruta et al., 2009; Xu et al., 2011; Challier et al., 2012; Perrier et al., 2014; Zhao Q. et al., 2014), one of which was capable of real-time determination of argininamide (Ozalp, 2012).

While medical diagnostics involve the detection and identification of diseases, therapy monitoring might be necessary for drugs that have a narrow therapeutic range, which means that the dose needed to gain a therapeutic effect may be relatively close to the toxic one. One example for this is digoxin, a cardiac glycoside that is used for congestive heart failure and atrial fibrillation. An aptamer binding to it has been selected and used to counter digoxin effects in guinea-pig atria, making it a possible treatment for digoxin poisoning, but it has not been implemented for the intended purpose of therapy monitoring (Kiani et al., 2012). Another drug with a narrow therapeutic range is theophylline. The bronchodilator is used for asthmatic conditions and very frequently clinically monitored. Several sensors using different theophylline aptamers were used to detect it in serum (Ferapontova et al., 2008; Sato et al., 2012; Dong and Zhao, 2013; Jiang et al., 2015). The same is true for aminoglycoside antibiotics: As overdosing can lead to hearing loss, tinnitus, and kidney failure, careful monitoring of their blood concentration is necessary. To this end, aptasensors have been developed for gentamicin (Rowe et al., 2010), tobramycin (González-Fernández et al., 2011; Cappi et al., 2015), kanamycin (Li et al., 2014), and streptomycin (Danesh et al., 2016; Emrani et al., 2016) detection in serum.

A microfluidic sensor that allows real-time monitoring of cocaine in undiluted blood serum was published. While the ability to monitor cocaine might not be in high demand, a similar sensor for drugs with a narrow therapeutic range such as those described above would probably be highly appreciated (Swensen et al., 2009).

Ibuprofen and diclofenac are part of a group of over-the-counter analgesic drugs that account for a large number of cases reported to the American Association of Poison Control Centers. Even though they do not suffer from a narrow therapeutic range, monitoring of the drug levels is necessary in cases of poisoning (White and Wong, 1998). For both ibuprofen and diclofenac, aptasensors were used to determine the drug concentrations in serum or blood samples (Kashefi-Kheyrabadi and Mehrgardi, 2012; Roushani and Shahdost-fard, 2015).

#### RNA-imaging

Although the first RNA and DNA aptamers recognizing small molecules were selected against organic dyes (Ellington and Szostak, 1990, 1992) and followed by several selections against different fluorophores, none of them was used for RNA-imaging (Holeman et al., 1998; Werstuck and Green, 1998; Wilson and Szostak, 1998). Aptamers that led to increased fluorescence intensity upon binding to their fluorophore target were used for DNA (Kolpashchikov, 2005) and RNA detection (Endo and Nakamura, 2010) and as a fluorescent tag for transcriptional monitoring (Sando et al., 2008), but only *in vitro*.

The breakthrough for RNA-imaging in living cells was the selection of RNA aptamers binding small molecule mimics of the GFP-fluorophore by Paige et al. (2011). The aptamer that bound a mimic of the EGFP-fluorophore, 3,5-difluoro-4-hydroxybenzylidene imidazolinone (DFHBI), was termed spinach due to the bright green fluorescence of DFHBI upon aptamer binding. It was used as a fluorescent tag for real time rRNA-imaging in mammalian cells (Paige et al., 2011). Since then, spinach was used for *in vitro* visualization of enzymatic RNA synthesis (Höfer et al., 2013), conformational transitions of a cyclic-di-GMP riboswitch (Luo et al., 2014b), and siRNA processing by dicer (Rogers et al., 2015). In combination with the expression of fluorescent proteins, RNA-spinach constructs allowed the visualization and quantification of transcription and translation in bacteria (Pothoulakis and Ellis, 2015) and artificial cells (van Nies et al., 2015).

Elaborate imaging techniques using spinach enabled the visualization of the response of a yeast cell to osmotic shock including induced transcription factors, target genes, and transcripts (Guet et al., 2015). The aptamer could be shown to have no influence on transcription, translation, and degradation of mRNAs and was further optimized for visualization of low abundant mRNAs in bacteria by increasing the number of aptamer copies (Zhang et al., 2015). An aptamer-variant of spinach, called brokkoli was used to optimize aptamer expression and stability in cells (Filonov et al., 2015). For the visualization of viral genome trafficking, a split version of spinach was introduced into viral particles (Tsvetkova et al., 2015).

Using a similar approach as for spinach, an aptamer called mango was selected, which binds to thiazole orange derivatives and has a higher affinity than spinach itself (3.2 instead of 300-540 nM). Its fluorescence could be detected intracellularly both after expression in bacteria and injection into *C. elegans* gonads. Mango was used for the visualization of bacterial 6S transcriptional control RNA (Dolgosheina et al., 2014).

Even without binding of spinach, DFHBI is slightly fluorescent. In an effort to establish a system with lower background fluorescence, tandem repeats of small molecule RNA aptamers were cloned behind a promotor of choice. Upon addition of Cy3- and Cy5-conjugated aptamer ligands, both bound to the aptamer repeats. The close proximity of the two fluorophores allowed the use of FRET as readout. The system was termed IMAGEtags and allowed live-cell imaging of transcription in real time (Shin et al., 2014).

A completely different approach was the selection of aptamers against quenchers, where binding of the aptamer inhibited quenching and thereby led to fluorescence emission (Sparano and Koide, 2005). The idea was used for real time detection of RNA transcripts *in vitro* (Murata et al., 2011), in bacterial cells (Sunbul and Jäschke, 2013), and for imaging of endogenous mRNAs in mammalian cells (Sato et al., 2015). An aptamer for the quencher dinitroaniline was combined with a sulforhodamine B aptamer to allow parallel imaging of two different RNAs in bacterial cells (Arora et al., 2015).

#### Allosteric modulation and photoregulation

While for most applications, the detection and quantification of the analyte by aptamer binding is sufficient, some require control over the binding event, e.g., because it needs to be switched on or off at a specific time point or location. A popular means of achieving spatiotemporal control is the use of light as a (dis)activator (You and Jaffrey, 2015). Aptamers have been selected against an azobenzene-containing peptide and a spiropyran molecule, both of which reversibly change their conformation between two isomers upon irradiation. The aptamers could be shown to selectively bind to one of the isomeric forms (Hayashi et al., 2007; Young and Deiters, 2008). Nonetheless, they have not been combined with e.g., a second aptamer to regulate its activity in a light-dependent manner.

A second possible trigger for the activation of aptamer binding is binding of a ligand to its aptamer, which thereupon changes its conformation and results in activation of a second part (Stojanovic and Kolpashchikov, 2004). Riboswitches follow this principle: binding of a specific metabolite leads to a conformational rearrangement causing regulation of transcription or translation (Breaker, 2012). Apart from the natural riboswitches, which are discussed in more detail below, artificial riboswitches, employing *in vitro* selected aptamers for small molecules as recognition elements, can be engineered to control gene expression (Schneider and Suess, 2016).

Werstuck and Green were the first to exploit the conformational change of an aptamer upon binding to its ligand, Hoechst dye 33258, to control gene expression in eukaryotic cells (Werstuck and Green, 1998). A different approach was chosen by Ausländer et al., who used theophylline to allosterically regulate an aptamer binding and inhibiting the TetR-protein, thereby regulating gene expression with the Tet Off system (Auslander et al., 2011).

Most allosteric uses of aptamers for small molecules are in the field of artificial ribozymes, where binding of the aptamer ligand leads to activation of the ribozyme and therefore cleavage of its target nucleic acid (Araki et al., 1998, 2001). Lee et al. combined this approach with light regulation: They selected an aptamer for the “closed” form of a photoswitchable dihydropyrene and connected it to the hammerhead ribozyme: Upon irradiation with UV-light, the ribozyme was activated whereas visible light stopped the cleavage reaction (Lee et al., 2007). Approaches where the aptamer is not used as regulator, but allosterically regulated by a different nucleic acid include an aptamer that only binds its target, ATP, in the presence of a specific mRNA (Cong and Nilsen-Hamilton, 2005) and complementary strands of different lengths that were used to fine-tune the affinity of a sensor based on the cocaine aptamer (Porchetta et al., 2012).

#### Riboswitches—natural aptasensors

Riboswitches are natural RNA-elements that consist of an aptamer domain and an expression platform. Binding of the specifically recognized metabolite causes a conformational change, which activates the expression platform. Depending on the riboswitch, this can lead to transcriptional down- or upregulation or translational inhibition of the mRNA that contains the riboswitch in its untranslated region (Breaker, 2012). Considering that their aptamer domain makes riboswitches natural small molecule aptasensors that allosterically modulate gene expression, it stands to reason to compare them to *in vitro* selected aptamers.

While an increasing number of small molecule aptamers with low nanomolar affinities is selected, many K_d_s are in the micrometer range (McKeague and Derosa, 2012 and Table 1). In contrast, seven out of 18 riboswitch classes reviewed by Lünse et al. can bind their targets with affinities in the single digit nM range and only six have micromolar affinities—all but one of which are in single digits. The exception is the glutamine riboswitch with a low affinity of at least 150 μM (Lünse et al., 2014).

This shows that at least RNA is capable of binding to small molecules with high affinities—a property that should be transferrable to aptamers. The only apparent difference between the two is the size as the aptamer domains in riboswitches are usually longer than selected aptamers and thereby allow more complex three-dimensional structures. A solution would be to select for aptamers with longer libraries. However, seeing as the amount of aptamers with high affinities is steadily increasing even without longer libraries, it is probably more a question of applying the correct selection pressure than a general—unsolvable—problem.

The other important property used to describe aptamers is selectivity. They can differentiate between very closely related molecules—the best example being the theophylline aptamer that discriminates by a factor of 10.000 between caffeine and theophylline, which differ by a single methyl group. However, said differentiation was already enforced during the selection process by deliberately removing sequences that also bind caffeine (Jenison et al., 1994). If such precautions are not taken, selectivity of the aptamer is left up to chance (Wallace and Schroeder, 1998).

Due to the fact that riboswitches are a common and essential mechanism for gene regulation in bacteria, they are nowadays recognized as a potential target for antibiotic compounds. Accordingly, research into analogs of the natural metabolite that also bind to the riboswitch and are therefore potential lead compounds for drug development has increased. As has been shown for aptamers, structural deviations of the ligand are indeed possible and do not necessarily result in a large decrease of affinity. Nonetheless, the sites responsible for the interaction between riboswitch and ligand have to remain unmodified (Matzner and Mayer, 2015). In the same manner, aptamers are only specific for those parts of the target molecule that they interact with—an interaction that can be enforced by counter selection with molecules that differ from the target in that specific position as described above.

## Conclusion

We have shown that a wide variety of aptamer-based sensors for a multitude of small molecule targets is already available. While some still lack sensitivity, a lot of them already reach the detection and quantification limits needed for the respective applications.

As small molecule sensors based on aptamers would be capable of solving important problems such as the detection of environmental and food contaminations, focusing on commercializing those sensors that are already available would not only be beneficial regarding those problems, but would also be another step toward proving the general usefulness of aptamers.

For a large number of interesting small molecule targets, no aptamers are available yet. While for some, it is just a matter of nobody having tried to select an aptamer yet, many targets are difficult to address. This might be because they are hard to immobilize or due to a very limited number of interaction possibilities with nucleic acids. Nevertheless, with the steady progress in the SELEX field that comes with new technologies like next generation sequencing and selection techniques such as capillary electrophoresis-SELEX, the fraction of non-addressable targets decreases. In addition, the finding that nucleobase-modifications can increase the success rate of aptamer selections for proteins immensely (Gold et al., 2010), should also be applicable to small molecule targets.

## Author contributions

FP and GM wrote the review.

### Conflict of interest statement

The authors declare that the research was conducted in the absence of any commercial or financial relationships that could be construed as a potential conflict of interest.

## Footnotes

^1^Pubmed search for “siRNA”: 72390 hits, “aptamer”: 5583 hits (02.01.2016)

^2^http://www.sekisuidiagnostics.com/products/722-thrombin-activity-assay (26.01.2016)

^3^http://www.sekisuidiagnostics.com/products/724-apc-activity-assay (26.01.2016)

^4^PDB search for “riboswitch”: 208 hits (22.03.2016)

^5^Dynabeads M-270 Epoxy (Invitrogen): 100–200 μmol/g active chemical functionality; usually used per round: 50 μl 10 mg/ml; overall available: 5–10 nmol chemical functionality. Epoxy-activated Sepharose 6B (GE Healthcare): 19–40 μmol/ml medium active chemical functionality; usually used per round: 50 μl; overall available: 0.95–2 μmol chemical functionality.

^6^http://www.fao.org/food/food-safety-quality/a-z-index/mycotoxins/en/ (14.12.2015)
